# Erdafitinib Resensitizes ABCB1-Overexpressing Multidrug-Resistant Cancer Cells to Cytotoxic Anticancer Drugs

**DOI:** 10.3390/cancers12061366

**Published:** 2020-05-26

**Authors:** Chung-Pu Wu, Tai-Ho Hung, Sung-Han Hsiao, Yang-Hui Huang, Lang-Cheng Hung, Yi-Jou Yu, Yu-Tzu Chang, Shun-Ping Wang, Yu-Shan Wu

**Affiliations:** 1 Graduate Institute of Biomedical Sciences, College of Medicine, Chang Gung University, Tao-Yuan 33302, Taiwan; wuchung@mail.cgu.edu.tw (C.-P.W.); johnson170_ya@hotmail.com (S.-H.H.); yanghui.huang01@gmail.com (Y.-H.H.); ttpss91192@gmail.com (Y.-J.Y.); yyya.tw@yahoo.com.tw (Y.-T.C.); 2 Department of Physiology and Pharmacology, College of Medicine, Chang Gung University, Tao-Yuan 33302, Taiwan; 3 Department of Obstetrics and Gynecology, Taipei Chang Gung Memorial Hospital, Taipei 10507, Taiwan; thh20@adm.cgmh.org.tw; 4 Department of Chinese Medicine, College of Medicine, Chang Gung University, Tao-Yuan 33302, Taiwan; 5 Department of Chemistry, Tunghai University, Taichung 40704, Taiwan; oscarhung19000@gmail.com; 6 Department of Orthopedics, Taichung Veterans General Hospital, Taichung 40705, Taiwan; wsp0120@yahoo.com.tw; 7 Sports Recreation and Health Management Continuing Studies-Bachelor’s Degree Completion Program, Tunghai University, Taichung 40704, Taiwan

**Keywords:** chemoresistance, combination chemotherapy with JNJ-42756493, drug repositioning, modulator, P-glycoprotein

## Abstract

The development of multidrug resistance (MDR) in cancer patients, which is often associated with the overexpression of ABCB1 (MDR1, P-glycoprotein) in cancer cells, remains a significant problem in cancer chemotherapy. ABCB1 is one of the major adenosine triphosphate (ATP)-binding cassette (ABC) transporters that can actively efflux a range of anticancer drugs out of cancer cells, causing MDR. Given the lack of Food and Drug Administration (FDA)-approved treatment for multidrug-resistant cancers, we explored the prospect of repurposing erdafitinib, the first fibroblast growth factor receptor (FGFR) kinase inhibitor approved by the FDA, to reverse MDR mediated by ABCB1. We discovered that by reducing the function of ABCB1, erdafitinib significantly resensitized ABCB1-overexpressing multidrug-resistant cancer cells to therapeutic drugs at sub-toxic concentrations. Results of erdafitinib-stimulated ABCB1 ATPase activity and in silico docking analysis of erdafitinib binding to the substrate-binding pocket of ABCB1 further support the interaction between erdafitinib and ABCB1. Moreover, our data suggest that ABCB1 is not a major mechanism of resistance to erdafitinib in cancer cells. In conclusion, we revealed an additional action of erdafitinib as a potential treatment option for multidrug-resistant cancers, which should be evaluated in future drug combination trials.

## 1. Introduction

Several members of the human adenosine triphosphate (ATP)-binding cassette (ABC) transporter superfamily are linked to cancer multidrug resistance (MDR), which is one of the most difficult challenges in cancer chemotherapy [1,2]. ABCB1 (MDR1; P-glycoprotein) and ABCG2 (BCRP; MXR) are two of the most studied and well-characterized ABC drug transporters, capable of using chemical energy derived from ATP hydrolysis to transport cytotoxic anticancer drugs such as anthracyclines, paclitaxel, SN-38, topotecan, and Vinca alkaloids out of cancer cells. Consequently, the decreased intracellular drug accumulation in cancer cells leads to reduced drug sensitivity, treatment failure, and relapse in cancer patients [2,3,4]. Notably, many studies have reported that high expression of ABCB1 and/or ABCG2 is associated with MDR [1,2] and poor clinical outcome in patients with metastatic breast cancer (MBC) [5], blood cancers such as acute lymphocytic leukemia (ALL), acute myelogenous leukemia (AML), chronic lymphocytic leukemia (CLL) [6,7,8,9], and multiple myeloma (MM) [10,11,12]. Therefore, it is of great clinical significance to develop therapeutic agents against the activity of ABCB1 and/or ABCG2.

Despite a large number of different classes of novel inhibitors of ABCB1 and/or ABCG2 that have been developed over the years, there are currently no U.S. Food and Drug Administration (FDA)-approved therapeutic agents that can be used to deal with MDR in patients, mostly due to the lack of selectivity and adverse drug–drug interactions [2,13,14,15,16]. Therefore, instead of synthesizing novel modulators, an alternative approach is to explore the possibility of repurposing FDA-approved tyrosine kinase inhibitors (TKIs) to resensitize ABCB1- or ABCG2-overexpressing multidrug-resistant cancer cells to chemotherapeutic agents [17,18]. Erdafitinib is a small-molecule pan-fibroblast growth factor receptor (FGFR) kinase inhibitor that binds and inhibits the activity of FGFR1–4 and the proliferation of cancer cells [19]. It is the first FGFR kinase inhibitor approved by the FDA for the treatment of adult patients with locally advanced or metastatic urothelial carcinoma (mUC), based on data from a trial of erdafitinib in participants with urothelial cancer (ClinicalTrials.gov Identifier: NCT02365597). In addition, erdafitinib is currently being evaluated as monotherapy or combination therapy in clinical trials for patients with advanced solid tumors with FGFR alterations (NCT04083976), metastatic or locally advanced urothelial cancer with selected FGFR gene alterations (NCT03473743), relapsed or refractory advanced solid tumors, non-Hodgkin lymphoma, or histiocytic disorders with FGFR mutations (NCT03210714), double negative prostate cancer (NCT03999515), non-small-cell lung cancer (NSCLC), urothelial cancer, esophageal cancer, and cholangiocarcinoma (NCT02699606), ER+/HER2-/FGFR-amplified metastatic breast cancer (NCT03238196), and advanced hepatocellular carcinoma (HCC) (NCT02421185).

In the present work, the potential chemosensitization effect of erdafitinib on multidrug-resistant cancer cells was assessed in human cell lines overexpressing ABCB1 or ABCG2. Our data indicate that erdafitinib is equally effective in treating ABCB1- and ABCG2-overexpressing multidrug-resistant cancer cells as drug-sensitive cancer cells. More importantly, we discovered an additional mode of action of erdafitinib on modulating the transport function of ABCB1 and resensitizing ABCB1-overexpressing multidrug-resistant cancer cells to conventional chemotherapeutic drugs.

## 2. Results

### 2.1. Cells Overexpressing ABCB1 or ABCG2 Are Not Resistant to Erdafitinib

Previous reports have demonstrated that ABCB1 and ABCG2 are capable of effluxing small molecule inhibitors of receptor tyrosine kinases (RTKs) such as sunitinib [20,21] and imatinib [22,23,24,25] out of cancer cells. Consequently, ABCB1- and ABCG2-overexpressing cancer cells are less susceptible to treatment with these RTKs inhibitors [26,27,28]. To this end, the cytotoxicity of erdafitinib was determined in drug-sensitive human cancer cells and the ABCB1- or ABCG2-overexpressing multidrug-resistant variants as well as in HEK293 cells and HEK293 cells stably transfected with human ABCB1 or ABCG2. As shown in Table 1, the ABCB1-overexpressing multidrug-resistant KB-V-1 human epidermal cancer cells, NCI-ADR-RES human ovarian cancer cells, ABCB1-transfected MDR19-HEK293 cells, and the corresponding drug-sensitive parental KB-3-1, OVCAR-8, and pcDNA-HEK293 cells are equally sensitive to erdafitinib treatment. Similarly, the ABCG2-overexpressing multidrug-resistant H460-MX20 human lung cancer cells, S1-M1-80 human colon cancer cells, ABCG2-transfected R482-HEK293 cells, and the corresponding drug-sensitive parental H460, S1 and pcDNA-HEK293 cells are also equally sensitive to erdafitinib treatment.

### 2.2. Erdafitinib Reverses Multidrug Resistance Mediated by ABCB1

**Figure 1:**
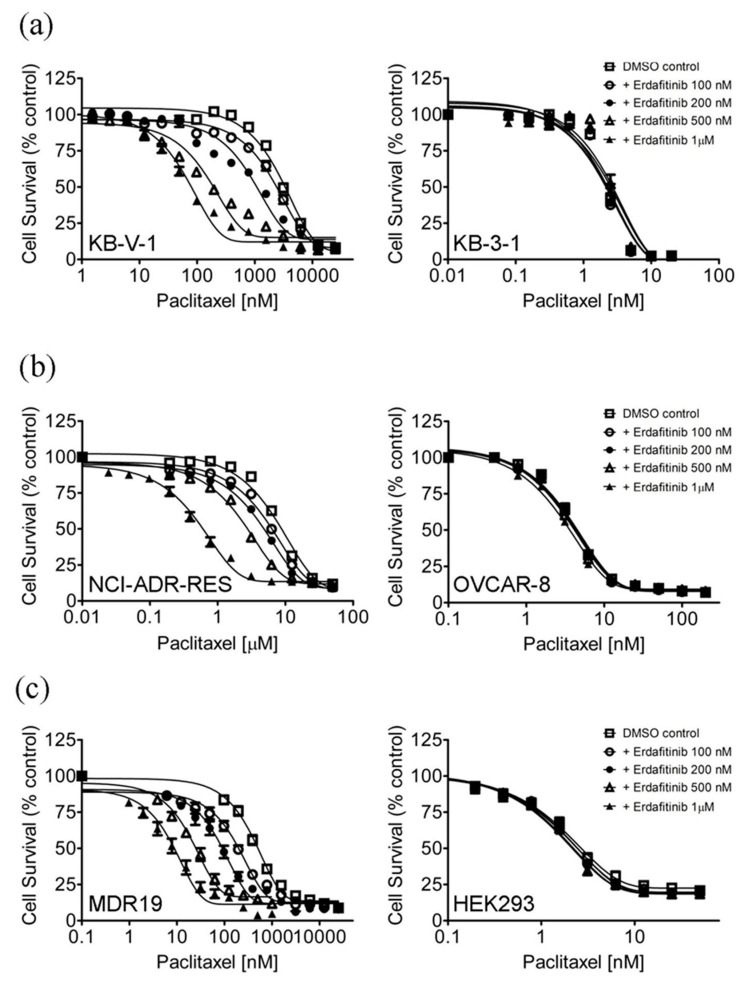
Erdafitinib sensitizes ABCB1-overexpressing multidrug-resistant cells to the ABCB1 substrate drug paclitaxel. The effect of erdafitinib on ABCB1-mediated paclitaxel resistance was examined in (**a**) ABCB1-overexpressing multidrug-resistant KB-V-1 human epidermal cancer cells (left panel) and the corresponding drug-sensitive parental KB-3-1 cancer cells (right panel), (**b**) ABCB1-overexpressing NCI-ADR-RES human ovarian cancer cells (left panel) and the corresponding drug-sensitive parental OVCAR-8 cancer cells (right panel) as well as (**c**) HEK293 cells transfected with human ABCB1 (MDR1, left panel) and parental HEK293 cells (right panel). Cells were treated with increasing concentrations of paclitaxel, a well-known substrate of ABCB1, in the presence of DMSO (open squares) or erdafitinib at 100 nM (open circles), 200 nM (filled circles), 500 nM (open triangles), or at 1.0 μM (filled triangles). Points, mean values from at least three independent experiments; bars; S.E.M.

Several receptor tyrosine kinase inhibitors (RTKIs) have been reported to interact strongly with ABCB1 and/or ABCG2 and reverse multidrug resistance in cancer cells overexpressing ABCB1 and/or ABCG2 [17,18,29]. Therefore, the effect of erdafitinib on multidrug resistance mediated by ABCB1 and ABCG2 was determined. First, without significantly affecting the drug-sensitive parental cells, the chemosensitivity of ABCB1-overexpressing multidrug-resistant KB-V-1 (Figure 1a), NCI-ADR-RES (Figure 1b), and ABCB1-transfected MDR19-HEK293 cells (Figure 1c) to paclitaxel, a known substrate for ABCB1 [30], was restored by erdafitinib in a concentration-dependent manner. Similarly, erdafitinib reversed ABCB1-mediated resistance to vincristine, another well-known substrate of ABCB1 [30], in these multidrug-resistant cell lines (Table 2). In contrast, erdafitinib had no significant effect on ABCG2-mediated resistance to topotecan or SN-38, known substrates for ABCG2 [31,32], in ABCG2-overexpressing multidrug-resistant H460-MX20, S1-M1-80, and ABCG2-transfected R482-HEK293 cells (Table 3). Of note, verapamil and Ko143 were used as reference inhibitors for ABCB1 and ABCG2, respectively. The extent of chemosensitization by erdafitinib or verapamil or Ko143 in a particular cell line is represented by the fold-reversal (FR) value, calculated by dividing the IC_50_ value of a particular drug substrate by the IC_50_ value of the drug substrate in the presence of erdafitinib or verapamil or Ko143, as described previously [33]. Our results revealed that at sub-toxic concentrations, erdafitinib selectively reverses multidrug resistance mediated by ABCB1 in multidrug-resistant human cancer cell lines.

### 2.3. Erdafitinib Restores the Intracellular Drug Accumulation in ABCB1-Overexpressing Cells

**Figure 2:**
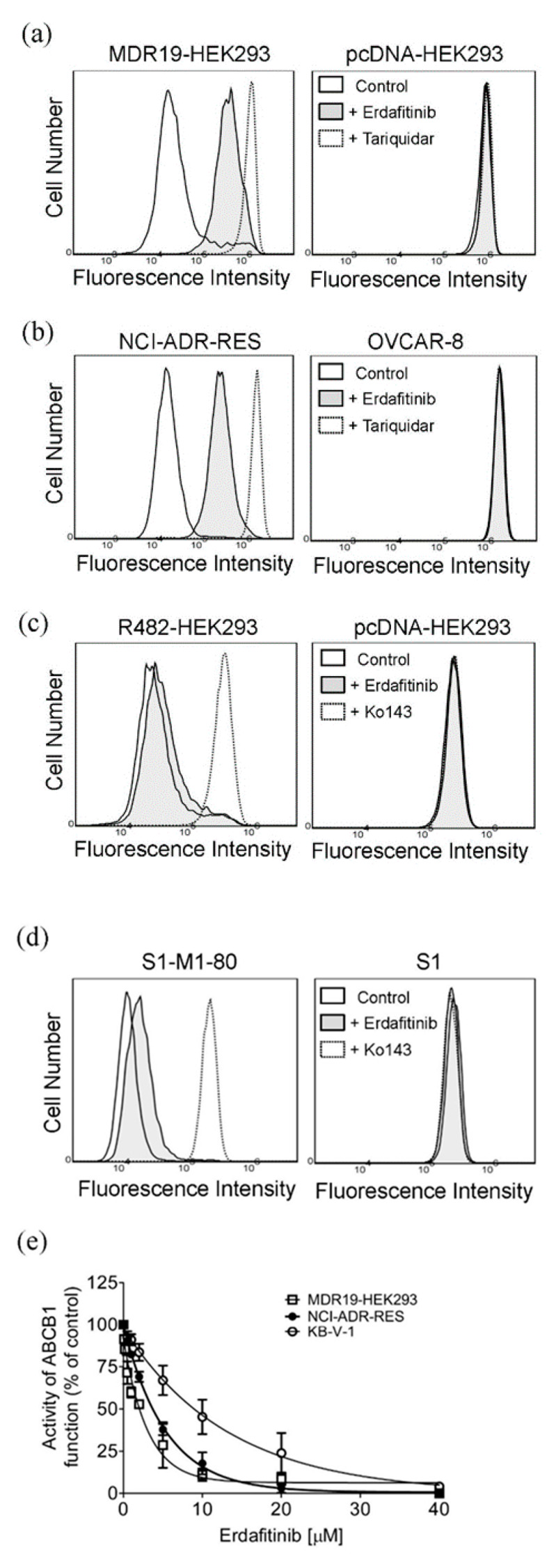
Erdafitinib increases the intracellular accumulation of ABCB1 fluorescent substrate drug in ABCB1-overexpressing cells. The intracellular accumulation of calcein, a fluorescent product of a known ABCB1 substrate calcein-AM, was determined in the (**a**) ABCB1-transfected MDR19-HEK293 cells (left panel) and the corresponding drug-sensitive parental HEK293 cells (right panel), and the (**b**) ABCB1-overexpressing NCI-ADR-RES cancer cells (left panel) and the corresponding drug-sensitive parental OVCAR-8 cancer cells (right panel), whereas the intracellular accumulation of pheophorbide A (PhA), a known ABCG2 fluorescent substrate drug, was determined in (**c**) ABCG2-transfected R482-HEK293 cells (left panel) and the corresponding drug-sensitive parental HEK293 cells (right panel), and the (**d**) ABCG2-overexpressing S1-M1-80 cancer cells (left panel) and the corresponding drug-sensitive parental S1 cancer cells (right panel). Cells were treated with DMSO (solid line), 1 μM of erdafitinib (filled solid line) or a reference inhibitor (dotted line) for ABCB1 (3 μM tariquidar, **a**,**b**) or for ABCG2 (1 μM Ko143, **c**,**d**) as a positive control. The respective fluorescent signals were analyzed by flow cytometry as described previously [39]. Representative histograms of at least three independent experiments are shown. (**e**) Concentration-dependent inhibition of ABCB1-mediated calcein efflux in MDR19-HEK293 cells (open squares), NCI-ADR-RES (filled circles) and KB-V-1 cancer cells (open circles). Points, mean values from at least three independent experiments; bars; S.D.

**Figure 3:**
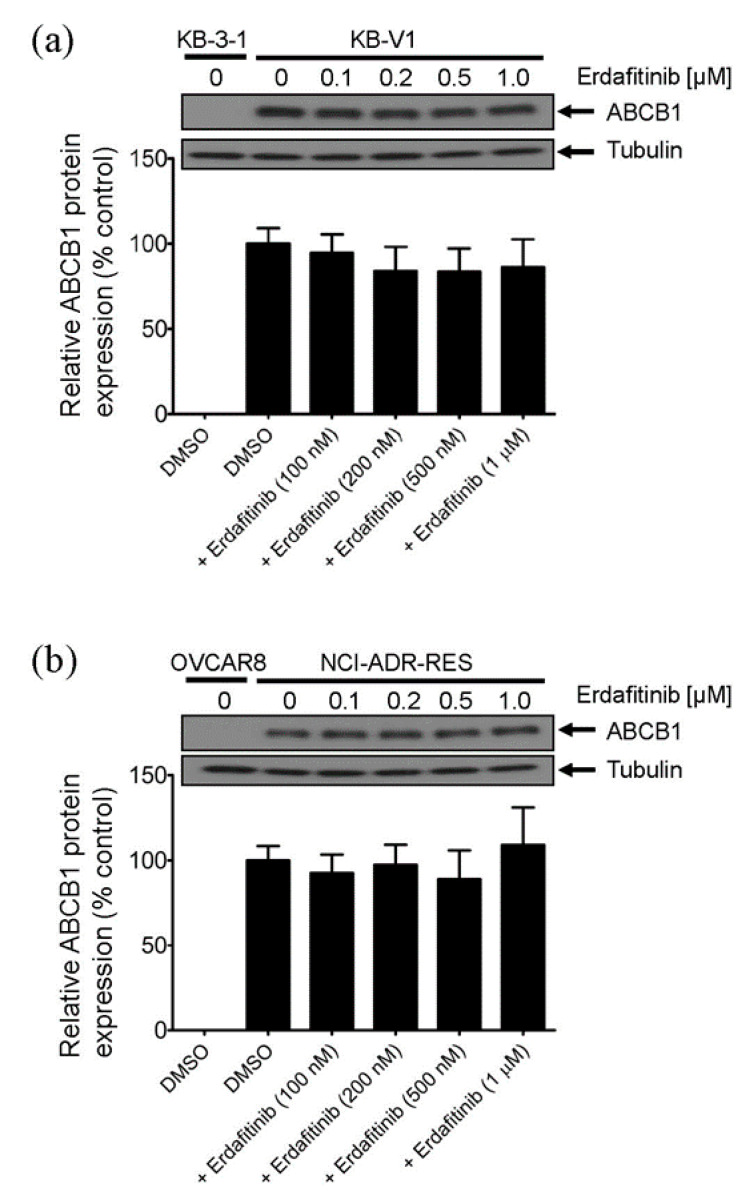
Erdafitinib does not alter the protein expression of ABCB1 in ABCB1-overexpressing cancer cells. (**a**) KB-V-1 cancer cells and (**b**) NCI-ADR-RES cancer cells were treated with DMSO (vehicle control) or erdafitinib at 100 nM, 200 nM, 500 nM, or 1 μM for 72 h and processed for western blotting as described in the Materials and Methods. α-tubulin was used as an internal loading control. The representative immunoblots (upper panel) and the corresponding quantification (lower panel) human ABCB1 protein are shown. Values are presented as mean ± S.D. calculated from at least three independent experiments. Detailed information about western blot can be found in Figures S1 and S2.

Direct inhibition of the drug efflux function and transient downregulation of drug transporters [34,35,36] are two of the most common ways for a modulator to resensitize multidrug-resistant cancer cells to chemotherapeutic drugs. To this end, the effect of erdafitinib on ABCB1-mediated efflux of calcein, a fluorescent product of an ABCB1 substrate calcein-AM [37], and ABCG2-mediated efflux of Pheophorbide A (PhA), a fluorescent substrate of ABCG2 [38], was determined in cells overexpressing ABCB1 or ABCG2. Erdafitinib at 1 μM significantly increased the intracellular accumulation of calcein in MDR19-HEK293 cells (Figure 2a) and NCI-ADR-RES cells (Figure 2b). In contrast, erdafitinib had minimal effect on the intracellular accumulation of PhA in R482-HEK293 cells (Figure 2c) and S1-M1-80 cells (Figure 2d). Of note, tariquidar at 3 μM was used as a reference inhibitor of ABCB1, whereas Ko143 at 1 μM was used as a reference inhibitor of ABCG2. Erdafitinib, tariquidar, and Ko143 had no significant effect on the intracellular drug accumulation in parental cell lines (Figure 2a–d, right panels). Moreover, erdafitinib inhibited ABCB1-mediated calcein efflux in a concentration-dependent manner in MDR19-HEK293 cells, NCI-ADR-RES, and KB-V-1 cells (Figure 2e) with a calculated IC_50_ value of approximately 1.8 μM, 3.6 μM, and 9.3 μM, respectively. Next, the potential effect of erdafitinib on the protein expression of ABCB1 was examined in ABCB1-overexpressing NCI-ADR-RES and KB-V-1 cancer cells. Cells were treated with increasing concentrations of erdafitinib (0.1–1.0 μM) for 72 h followed by immunoblotting, as described in the Materials and Methods. As shown in Figure 3a,b, erdafitinib had no significant effect on the protein expression of ABCB1 in either NCI-ADR-RES or KB-V-1 cancer cell lines. Our data suggest that erdafitinib reverses multidrug resistance by inhibiting the drug transport function of ABCB1, and not by downregulating the ABCB1 protein in ABCB1-overexpressing cancer cell lines.

### 2.4. Erdafitinib Restores the Effect of Drug-Induced Apoptosis in ABCB1-Overexpressing Multidrug-Resistant Cancer Cells

**Figure 4:**
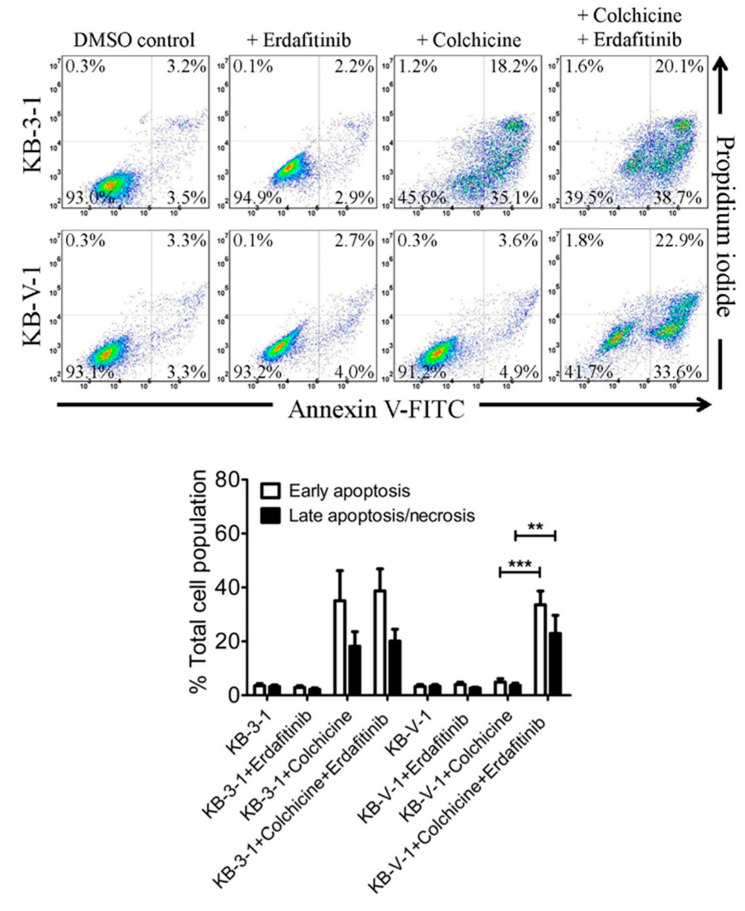
Erdafitinib sensitizes ABCB1-overexpressing multidrug-resistant cancer cells to apoptosis induction by an ABCB1 substrate drug. The representative flow cytometric dot plots (upper panel) and quantification (lower panel) of drug-sensitive KB-3-1 cells and the ABCB1-overexpressing multidrug resistant KB-V-1 cells treated with either DMSO (control), 5 μM of erdafitinib (+erdafitinib), 500 nM of colchicine (+colchicine), or a combination of colchicine and erdafitinib (+colchicine +erdafitinib). Cells were treated with the respective drug regimens and analyzed by flow cytometry as described in the Materials and Methods. The corresponding quantification data are presented as the mean ± SD calculated from at least three independent experiments. ** *p* < 0.01; *** *p* < 0.001, versus the same treatment in the absence of erdafitinib.

To confirm that erdafitinib reverses ABCB1-mediated multidrug resistance by restoring the cytotoxicity of cytotoxic drugs in ABCB1-overexpressing cancer cells and not by initiating a growth retardation effect, the effect of erdafitinib on drug-induced apoptosis in ABCB1-overexpressing cancer cells was examined. Drug-sensitive parental KB-3-1 and ABCB1-overexpressing KB-V-1 cancer cells were treated with 0.5 μM of colchicine, a known transported substrate of ABCB1 [30] and a known inducer of apoptosis [40], in the presence or absence of 5 μM of erdafitinib for 48 h before processed as detailed in the Materials and Methods. As shown in Figure 4, colchicine substantially induced the level of apoptosis in KB-3-1 cells (from an approximately 7% basal level to 53% of total apoptosis), but not in KB-V-1 cells (from approximately 7% basal level to 9% of total apoptosis). While treatment with erdafitinib alone did not induce significant apoptosis in KB-3-1 and KB-V-1 cells, the colchicine-induced apoptosis in KB-V-1 cells was significantly increased by erdafitinib from 9% to 57% of early and late apoptosis.

### 2.5. Erdafitinib Stimulates the ATPase Activity of ABCB1

**Figure 5:**
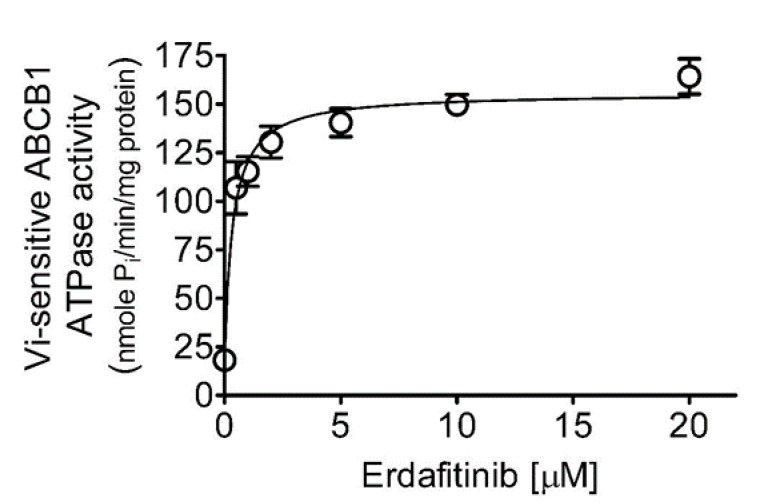
Erdafitinib stimulates ABCB1 ATPase activity. The effect of erdafitinib on vanadate-sensitive ATPase activity of ABCB1 was determined by the endpoint P_i_ assay as described in the Materials and Methods. Data are presented as a mean ± S.D. from three independent experiments.

Knowing that drug transport mediated by ABCB1 is coupled to ABCB1-mediated ATP hydrolysis [41,42], the effect of erdafitinib on vanadate (Vi)-sensitive ATPase activity of ABCB1 was examined. As shown in Figure 5, erdafitinib stimulated ABCB1 ATPase activity in a concentration-dependent manner with a 9-fold maximum stimulation and with the half-maximal effective concentration (EC_50_) value of approximately 0.35 μM (basal, 18.11 ± 2.08 nmoles P_i_/min/mg protein).

### 2.6. Docking Analysis of Erdafitinib with The Structure of ABCB1

**Figure 6:**
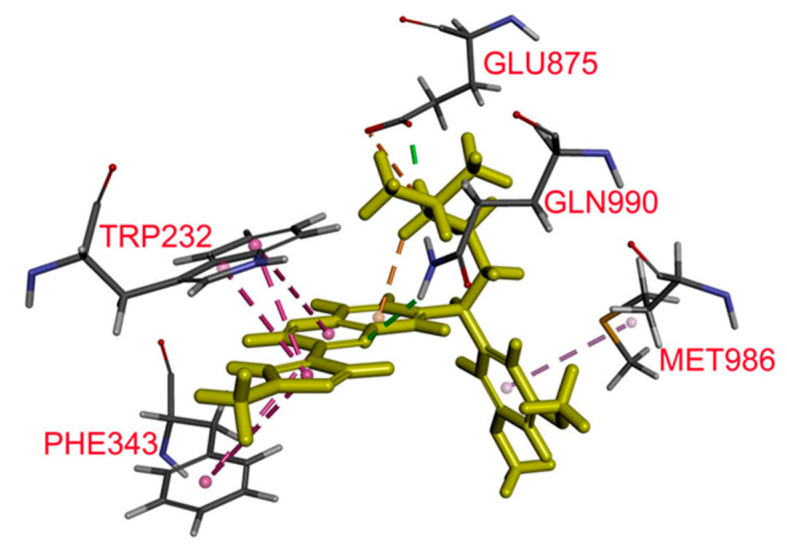
Docking of erdafitinib in the drug-binding pocket of ABCB1. Binding modes of erdafitinib with ABCB1 protein structure (PDB: 6QEX) was predicted by BIOVIA (Accelrys) Discovery Studio 4.0 software (San Diego, CA, USA) as described in the Materials and Methods. Erdafitinib is shown as a molecular model with the highlighted yellow color and the atoms for interacting amino acid residues are colored as carbon, gray; hydrogen, light gray; nitrogen, blue; and oxygen, red. Dotted lines indicate proposed interactions.

The most energy favorable docking pose of erdafitinib with the ABCB1 protein structure showed five probable interactions of erdafitinib with amino acids on transmembrane helices. The protonated nitrogen at the isopropyl amine moiety of erdafitinib was predicted to interact with Glu^875^ by both hydrogen bonding and charge–charge interaction. Another hydrogen bond was predicted between the protonated amide on the side chain of Gln^990^ with nitrogen on the quinoxaline ring. Phe^343^, Trp^232^, and Met^986^ were predicted to interact with aromatic rings on erdafitinib via hydrophobic interaction (Figure 6).

## 3. Discussion

Overexpression of ABCB1 in cancer cells is one of the known factors associated with poor therapeutic response in patients receiving cytotoxic anticancer agents [2,43]. One of the reasons why ABCB1-mediated multidrug resistance remains a substantial challenge for oncologists is due to the lack of clinically active inhibitors for the treatment of multidrug-resistant cancers [2]. Despite the substantial efforts that have been invested by drug developers, problems associated with the lack of selectivity and adverse drug–drug interactions have hindered the development of novel synthetic inhibitors of ABCB1 [16]. For instance, previous clinical trials of tariquidar (XR9576) in combination with vinorelbine (NCT00042315) or paclitaxel (NCT00042302) to treat non-small cell lung cancer (NSCLC) were terminated prematurely due to toxicity. In contrast, recent studies have demonstrated that several FDA-approved therapeutic agents such as osimertinib and midostaurin exhibited an inhibitory effect on ABCB1-mediated drug efflux, thus resensitizing ABCB1-overexpressing multidrug-resistant cancer cells to cytotoxic anticancer agents [29,44,45,46]. Moreover, the advantage of using combination therapy against multidrug-resistant cancers was demonstrated in a recent phase I study of concomitant administration of doxorubicin with nilotinib as a co-adjuvant treatment in sarcoma patients [47]. Similarly, studies have reported that a combination-therapy of lapatinib and capecitabine was superior to capecitabine alone in patients with human epidermal growth factor receptor 2 (HER2)-positive advanced breast cancer [48,49], whereas erlotinib significantly improved the survival of patients with advanced pancreatic cancer receiving gemcitabine [50,51]. These findings prompted us to investigate the chemosensitization effect of erdafitinib, the first FDA-approved FGFR kinase inhibitor, in ABCB1-overexpressing multidrug-resistant cancer cells.

The in vitro antiproliferative activity of erdafitinib has been shown in a large panel of human cancer cell lines of diverse tissue origins, with IC_50_ values ranging from below 1 µM to over 5 µM, dependent on the expression of FGFR in these cell lines [19]. However, the effect of erdafitinib against human multidrug-resistant cancer cells was not determined. Therefore, we first examined the cytotoxicity of erdafitinib in multiple human multidrug-resistant cancer cell lines that overexpress ABCB1 or ABCG2 as well as in the corresponding drug-sensitive parental cancer cell lines. We discovered that unlike other ABCB1- and ABCG2-interacting tyrosine kinase inhibitors such as dasatinib [28] and imatinib [26], erdafitinib was equally cytotoxic in multidrug-resistant cancer cells overexpressing either ABCB1 or ABCG2 as in the corresponding drug-sensitive parental cancer cells (Table 1). Moreover, it is worth noting that erdafitinib did not alter the protein expression of ABCB1 in KB-V-1 or NCI-ADR-RES cancer cells, suggesting that ABCB1 may not be a major contributor to the development of erdafitinib resistance in cancer patients. Nevertheless, the effect of regular treatment with erdafitinib on ABCB1 and ABCG2 in cancer patients remains to be further investigated.

**Figure 7:**
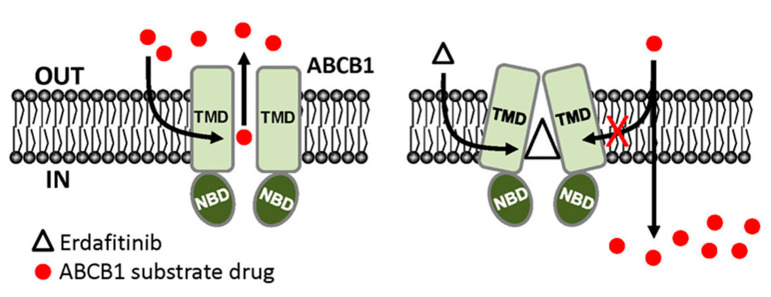
Simplified schematic diagram of erdafitinib modulating the drug efflux function of ABCB1 in multidrug-resistant cancer cells. In the absence of erdafitinib (white triangle), ABCB1 substrate chemotherapy drugs (red circles) are effluxed out of multidrug-resistant cancer cells by ABCB1, reducing the efficacy of these drugs. In contrast, binding of erdafitinib to the drug-binding pocket of ABCB1 outcompetes the binding of ABCB1 substrate chemotherapy drugs to the same site, consequently increasing the intracellular accumulation and efficacy of these drugs in ABCB1-overexpressing multidrug-resistant cancer cells.

Next, we examined the chemosensitizing effect of erdafitinib in multidrug-resistant cancer cells overexpressing ABCB1 or ABCG2. Results showed that erdafitinib reverses multidrug resistance mediated by ABCB1 (Table 2), but not by ABCG2 (Table 3), in human cancer cell lines at subtoxic concentrations (<1 µM) and in a concentration-dependent manner. Of note, the cytotoxicity of vincristine amplified by verapamil in drug-sensitive OVCAR-8 and KB-3-1 cancer cells (Table 2) was consistent with previous reports describing an increased cytotoxic effect of vincristine by verapamil at non-toxic concentrations [52,53]. Moreover, the chemosensitization effect of Ko143 on H460 cancer cells shown in Table 3 was due to the presence of basal expression of ABCG2 in H460 cancer cells [54,55]. More significantly, results of erdafitinib inhibiting the drug efflux function of ABCB1 (Figure 2) and improving the susceptibility of KB-V-1 cells to drug-induced apoptosis (Figure 4) indicate that erdafitinib reverses ABCB1-mediated multidrug resistance by restoring the cytotoxicity of ABCB1 substrate anticancer drugs in these multidrug-resistant cancer cells. Moreover, ABCB1-mediated ATP hydrolysis was stimulated by erdafitinib in a concentration-dependent manner (Figure 5), which is consistent with previous reports [29,33,46,56,57] where the ATPase activity of ABCB1 can be stimulated by the presence of a competitive inhibitor in the substrate-binding pocket of ABCB1. Together with the results of erdafitinib stimulating ABCB1 ATPase activity, the in silico docking analysis of erdafitinib binding to ABCB1 in the inward-open conformation (Figure 6) supports the notion that erdafitinib interacts with several amino acid residues within the transmembrane domain of ABCB1 and attenuates the binding of another drug substrate by direct competition (Figure 7). It is worth mentioning that platinum-based combination chemotherapy consisting of methotrexate, vinblastine, doxorubicin, and cisplatin remains one of the standard treatment options for metastatic urothelial carcinoma. Knowing that that methotrexate, vinblastine, and doxorubicin are substrates for ABCB1, it would be beneficial to include erdafitinib in combination therapy for metastatic urothelial carcinoma. Moreover, in the event that there are tumors harboring FGFR alterations and ABCB1 overexpression, erdafitinib would remain effective against FGFR, as we have shown that ABCB1 does not confer significant resistance to erdafitinib in cancer cells (Table 1).

## 4. Materials and Methods

### 4.1. Chemicals

Erdafitinib (JNJ-42756493) was purchased from Selleckchem (Houston, TX, USA). The Tools Cell Counting (CCK-8) Kit was obtained from Biotools Co. Ltd. (Taipei, Taiwan). All other chemicals were purchased from Sigma (St. Louis, MO, USA) unless stated otherwise. Dulbecco’s Modified Eagle’s Medium (DMEM), Iscove’s Modified Dulbecco’s Medium (IMDM), Rosewell Park Memorial Institute (RPMI) 1640 medium, fetal calf serum (FCS), phosphate-buffered saline (PBS), trypsin-EDTA, penicillin, and streptomycin were obtained from Gibco, Invitrogen (Carlsbad, CA, USA). The FITC Annexin V Apoptosis Detection Kit was purchased from BD Pharmingen (San Diego, CA, USA).

### 4.2. Cell Culture Conditions

The human embryonic kidney cell line HEK293, HEK293 cells stably transfected with human ABCB1 (MDR19-HEK293), and HEK293 cells stably transfected with wild-type human ABCG2 (R482-HEK293); the human epidermal cancer cell line KB-3-1; and its ABCB1-overexpressing variant KB-V-1 were maintained in DMEM supplemented with 10% FCS, l-glutamine and penicillin and streptomycin. The human ovarian cancer cell line OVCAR-8 and its ABCB1-overexpressing variant NCI-ADR-RES; the human non-small cell lung cancer (NSCLC) cell line H460 and its ABCG2-overexpressing variant H460-MX20; and the human colon cancer cell line S1 and its ABCG2-overexpressing variant S1-M1-80 were maintained in RPMI-1640 supplemented with 10% FCS, l-glutamine, and penicillin and streptomycin. HEK293 transfectants and KB-V-1 cells were maintained in the presence of 2 mg/mL of G418 [39] or 1 mg/mL of vinblastine [58], respectively. The S1-M1-80 and H460-MX20cells were maintained in the presence of 80 μM of mitoxantrone [59] or 20 nM of mitoxantrone [60], respectively. All cells were cultured at 37 °C in 5% CO_2_ humidified air and maintained in a drug-free medium for seven days before the assay.

### 4.3. Cell Viability Assay

The viability of cells treated with erdafitinib or other drug combinations was determined using the Cell Counting Kit-8 (CCK-8) assay or MTT assay based on the method described by Ishiyama et al. [61]. Briefly, cells were plated in 96-well flat-bottom plates and allowed to attach for 24 h at 37 °C in 5% CO_2_ humidified air, then incubated with erdafitinib or other drug combinations for an additional 72 h. At least three independent experiments were performed to obtain the IC_50_ values, calculated using the fitted concentration-response curve of each drug regimen. The extent of chemosensitization by a modulator was presented as a fold-reversal (FR) value, determined by adding a non-toxic concentration of erdafitinib or a reference inhibitor to the cytotoxicity assays as described previously [33,39].

### 4.4. Fluorescent Substrate Accumulation and Flow Cytometry Analysis

The intracellular drug accumulation experiments were carried out and analyzed according to the method described by Gribar et al. [62] using the CellQuest software and the FlowJo software (Tree Star, Inc., Ashland, OR, USA). The accumulation of the fluorescent ABCB1 substrate drug calcein [37] in ABCB1-overexpressing cells or the fluorescent ABCG2 substrate drug pheophorbide A (PhA) [38] in ABCG2-overexpressing cells was determined in the presence of DMSO (control), 1 μM of erdafitinib, 3 μM of tariquidar, or 1 μM of Ko143 using a Becton-Dickinson FACSort flow cytometer as described previously [38,63].

### 4.5. Immunoblotting

Sodium dodecyl sulfate-polyacrylamide gel electrophoresis (SDS–PAGE) and immunoblotting for ABCB1 and tubulin were performed as described previously [39]. ABCB1-overexpressing KB-V-1 and NCI-ADR-RES cancer cells were treated with DMSO (control) or erdafitinib at 100 nM, 200 nM, 500 nM, or 1μM for 72 h before being harvested and subjected to SDS-polyacrylamide electrophoresis and western blotting. The anti-P-glycoprotein antibody C219 (1:3000 dilution) and anti-alpha tubulin antibody (1:100,000 dilution) (Abcam, Cambridge, MA, USA) were used as the primary antibodies to detect ABCB1 and the positive loading control tubulin, respectively. The horseradish peroxidase-conjugated goat anti-mouse IgG (1:100,000 dilution) was used as the secondary antibody. The signals were detected and visualized using the enhanced chemiluminescence (ECL) kit (Merck Millipore, Billerica, MA, USA).

### 4.6. Apoptosis Assay

The effect of erdafitinib, alone or in combination with colchicine, a known apoptotic inducer, in drug-sensitive and ABCB1-overexpressing multidrug-resistant cancer cells was determined based on the concurrent annexin V–FITC and propidium iodide (PI) staining method, according to the manufacturer’s instructions (BD Pharmingen) and as previously described [64]. Briefly, drug-sensitive parental KB-3-1 and ABCB1-overexpressing KB-V-1 cells were treated with DMSO, erdafitinib alone, colchicine alone, or the combination of colchicine and erdafitinib as indicated for 48 h before stained with annexin V–FITC (1.25 µg/mL) and PI (0.1 mg/mL) for 15 min at room temperature. FACScan equipped with CellQuest software (Becton-Dickinson Biosciences, San Jose, CA, USA) was used to analyze the labeled cells as described previously [29].

### 4.7. ATPase Assay

The effect of erdafitinib on the vanadate (Vi)-sensitive ATPase activity of ABCB1 was determined based on the endpoint P_i_ assay [65] using the Pgp-Glo assay system (Promega, Madison, WI, USA) according to the manufacturer’s protocol and as previously described [66,67].

### 4.8. In Silico Docking of Erdafitinib in The Drug-Binding Pockets of ABCB1

The energy of the three-dimensional structure of ABCB1(PDB:6QEX) [68] was minimized using Acclerys Discovery Studio 4.0. The preparation of the structure of erdafitinib and docking was performed by the CDOCKER module of the same software.

### 4.9. Quantification and Statistical Analysis

The experimental data were expressed as mean ± standard deviation (S.D.) from at least three independent experiments unless stated otherwise. GraphPad Prism software (GraphPad Software, La Jolla, CA, USA) was used for curve plotting, and KaleidaGraph software (Synergy Software, Reading, PA, USA) was used for statistical analysis. The difference between the mean values of the experimental and control or improvement in fit was analyzed by the two-tailed Student’s t-test and labeled with asterisks as “statistically significant” if the probability, *p*, was less than 0.05.

## 5. Conclusions

In summary, without excluding the possibility that other mechanisms may also contribute to the reversal of multidrug resistance and that adverse drug reactions have been reported in combination therapy [69,70,71], our study demonstrated that erdafitinib is an FDA-approved therapeutic agent that could be utilized to fight multidrug-resistant cancers associated with the overexpression of ABCB1, and the concomitant administration of cytotoxic anticancer drugs with erdafitinib warrants further investigation.

## Figures and Tables

**Table 1:** Cytotoxicity of erdafitinib in human cell lines overexpressing ABCB1 or ABCG2.

Cell Line	Type	Transporter Expressed	IC_50_ (μM) ^†^
KB-3-1	epidermal	-	3.18 ± 0.48
KB-V-1	epidermal	ABCB1	4.92 ± 1.19
OVCAR-8	ovarian	-	10.80 ± 2.83
NCI-ADR-RES	ovarian	ABCB1	6.21 ± 1.15
H460	lung	-	3.46 ± 0.37
H460-MX20	lung	ABCG2	4.66 ± 0.71
S1	colon	-	4.31 ± 1.19
S1-M1-80	colon	ABCG2	5.60 ± 1.21
pcDNA-HEK293	-	-	6.02 ± 1.25
MDR19-HEK293	-	ABCB1	6.51 ± 1.27
R482-HEK293	-	ABCG2	5.16 ± 0.80

^†^ IC_50_ values are the mean ± SD calculated from dose-response curves obtained from at least three independent experiments using the cytotoxicity assay as described in the Materials and Methods.

**Table 2:** The effect of erdafitinib on ABCB1-mediated multidrug resistance in ABCB1-overexpressing human cell lines.

Treatment	Concentration (μM)	Mean IC_50_^†^ ± SD and (FR ^‡^)	
		OVCAR-8 (Parental) [nM]	NCI-ADR-RES (Resistant) [μM]
Paclitaxel	-	3.74 ± 0.67 (1.0)	7.96 ± 1.46 (1.0)
+ erdafitinib	0.1	3.57 ± 0.62 (1.0)	6.08 ± 0.88 (1.3)
+ erdafitinib	0.2	3.66 ± 0.63 (1.0)	4.76 ± 0.64 * (1.7)
+ erdafitinib	0.5	3.46 ± 0.63 (1.1)	2.80 ± 0.34 ** (2.8)
+ erdafitinib	1.0	3.00 ± 0.48 (1.2)	0.68 ± 0.10 *** (11.7)
+ verapamil	5.0	2.92 ± 0.55 (1.3)	0.31 ± 0.04 *** (25.7)
		[nM]	[μM]
Vincristine	-	69.30 ± 10.38 (1.0)	4.36 ± 0.96 (1.0)
+ erdafitinib	0.1	69.17 ± 12.60 (1.0)	3.41 ± 0.63 (1.3)
+ erdafitinib	0.2	64.38 ± 10.45 (1.1)	3.23 ± 0.61 (1.3)
+ erdafitinib	0.5	61.65 ± 11.86 (1.1)	1.73 ± 0.24 * (2.5)
+ erdafitinib	1.0	54.37 ± 9.90 (1.3)	0.57 ± 0.06 ** (7.6)
+ verapamil	5.0	19.20 ± 2.97 ** (3.6)	0.18 ± 0.04 ** (24.2)
**Treatment**	**Concentration** **(μM)**	**KB-3-1 (Parental)** **[nM]**	**KB-V-1 (Resistant)** **[μM]**
Paclitaxel	-	1.72 ± 0.66 (1.0)	2.84 ± 0.54 (1.0)
+ erdafitinib	0.1	1.71 ± 0.61 (1.0)	2.31 ± 0.20 (1.2)
+ erdafitinib	0.2	1.74 ± 0.63 (1.0)	1.11 ± 0.12 ** (2.6)
+ erdafitinib	0.5	2.16 ± 0.84 (0.8)	246.89 ± 39.24 ** [nM] (11.5)
+ erdafitinib	1.0	2.10 ± 0.81 (0.8)	83.93 ± 10.28 *** [nM] (33.9)
+ verapamil	5.0	1.71 ± 0.68 (1.0)	43.36 ± 5.94 *** [nM] (65.5)
		[nM]	[nM]
Vincristine	-	0.79 ± 0.30 (1.0)	954.77 ± 150.75 (1.0)
+ erdafitinib	0.1	0.76 ± 0.23 (1.0)	714.49 ± 172.29 (1.3)
+ erdafitinib	0.2	0.75 ± 0.23 (1.1)	341.99 ± 75.71 ** (2.8)
+ erdafitinib	0.5	0.80 ± 0.20 (1.0)	77.57 ± 19.24 *** (12.3)
+ erdafitinib	1.0	0.78 ± 0.18 (1.0)	20.47 ± 4.51 *** (46.6)
+ verapamil	5.0	0.15 ± 0.05 * (5.3)	8.74 ± 1.41 *** (109.2)
**Treatment**	**Concentration** **(μM)**	**pcDNA-HEK293** **(Parental)** **[nM]**	**MDR19-HEK293** **(Resistant)** **[nM]**
Paclitaxel	-	3.25 ± 0.69 (1.0)	532.02 ± 75.55 (1.0)
+ erdafitinib	0.1	2.72 ± 0.56 (1.2)	254.49 ± 41.07 ** (2.1)
+ erdafitinib	0.2	2.61 ± 0.53 (1.2)	110.74 ± 19.20 *** (4.8)
+ erdafitinib	0.5	2.35 ± 0.50 (1.4)	29.55 ± 5.21 *** (18.0)
+ erdafitinib	1.0	2.30 ± 0.50 (1.4)	10.10 ± 1.58 *** (52.7)
+ verapamil	5.0	2.13 ± 0.49 (1.5)	7.87 ± 1.71 *** (67.6)
		[nM]	[nM]
Vincristine	-	1.89 ± 0.23 (1.0)	363.39 ± 41.69 (1.0)
+ erdafitinib	0.1	1.99 ± 0.38 (0.9)	156.57 ± 21.81 ** (2.3)
+ erdafitinib	0.2	1.92 ± 0.38 (1.0)	95.18 ± 19.98 *** (3.8)
+ erdafitinib	0.5	1.74 ± 0.31 (1.1)	18.15 ± 2.53 *** (20.0)
+ erdafitinib	1.0	1.50 ± 0.30 (1.3)	5.59 ± 0.95 *** (65.0)
+ verapamil	5.0	0.65 ± 0.18 ** (2.9)	3.70 ± 0.57 *** (98.2)

FR, fold-reversal. ^†^ IC_50_ values are mean ± SD calculated from dose-response curves obtained from at least three independent experiments using cytotoxicity assay as described in the Materials and Methods. ^‡^ FR values were calculated by dividing the IC_50_ values of cells treated with a particular therapeutic drug in the absence of erdafitinib or verapamil by IC_50_ values of cells treated with the same therapeutic drug in the presence of erdafitinib or verapamil. * *p* < 0.05; ** *p* < 0.01; *** *p* < 0.001.

**Table 3:** The effect of erdafitinib on ABCG2-mediated multidrug resistance in ABCG2-overexpressing human cell lines.

Treatment	Concentration (μM)	Mean IC_50_^†^ ± SD and (FR ^‡^)	
		H460 (Parental) [nM]	H460-MX20 (Resistant) [μM]
Topotecan	-	64.14 ± 13.91 (1.0)	1.43 ± 0.26 (1.0)
+ erdafitinib	0.1	61.83 ± 13.83 (1.0)	1.29 ± 0.23 (1.1)
+ erdafitinib	0.2	61.07 ± 15.77 (1.0)	1.23 ± 0.22 (1.2)
+ erdafitinib	0.5	56.76 ± 12.36 (1.1)	1.10 ± 0.19 (1.3)
+ erdafitinib	1.0	46.20 ± 10.58 (1.4)	1.13 ± 0.21 (1.3)
+ Ko143	1.0	22.94 ± 5.53 ** (2.8)	99.10 ± 18.86 *** [nM] (14.4)
		[nM]	[nM]
SN-38	-	7.48 ± 1.36 (1.0)	126.16 ± 11.15 (1.0)
+ erdafitinib	0.1	7.54 ± 1.41 (1.0)	122.92 ± 11.13 (1.0)
+ erdafitinib	0.2	7.24 ± 1.37 (1.0)	120.46 ± 10.80 (1.0)
+ erdafitinib	0.5	6.40 ± 1.21 (1.2)	110.08 ± 10.57 (1.1)
+ erdafitinib	1.0	5.56 ± 1.13 (1.3)	100.69 ± 12.37 (1.3)
+ Ko143	1.0	3.19 ± 0.91 * (2.3)	6.79 ± 1.60 *** (18.6)
**Treatment**	**Concentration** **(μM)**	**S1 (Parental)** **[nM]**	**S1-M1-80 (Resistant)** **[μM]**
Topotecan	-	19.51 ± 2.34 (1.0)	6.35 ± 1.14 (1.0)
+ erdafitinib	0.1	20.40 ± 2.54 (1.0)	8.00 ± 1.60 (0.8)
+ erdafitinib	0.2	21.80 ± 2.96 (0.9)	6.25 ± 1.11 (1.0)
+ erdafitinib	0.5	22.31 ± 2.96 (0.9)	6.25 ± 1.50 (1.0)
+ erdafitinib	1.0	23.90 ± 3.55 (0.8)	6.65 ± 1.53 (1.0)
+ Ko143	1.0	20.88 ± 2.52 (0.9)	0.19 ± 0.05 *** (33.4)
		[nM]	[nM]
SN-38	-	3.03 ± 0.23 (1.0)	1036.58 ± 119.08 (1.0)
+ erdafitinib	0.1	3.07 ± 0.34 (1.0)	1083.89 ± 94.65 (1.0)
+ erdafitinib	0.2	3.23 ± 0.31 (0.9)	1397.68 ± 195.28 (0.7)
+ erdafitinib	0.5	3.37 ± 0.34 (0.9)	1251.31 ± 108.50 (0.8)
+ erdafitinib	1.0	2.76 ± 0.21 (1.1)	1128.26 ± 228.23 (0.9)
+ Ko143	1.0	2.64 ± 0.19 (1.1)	42.43 ± 9.66 *** (24.4)
**Treatment**	**Concentration** **(μM)**	**pcDNA-HEK293 (Parental)** **[nM]**	**R482-HEK293 (Resistant)** **[nM]**
Topotecan	-	25.56 ± 6.29 (1.0)	295.26 ± 25.68 (1.0)
+ erdafitinib	0.1	26.19 ± 6.47 (1.0)	274.81 ± 25.32 (1.1)
+ erdafitinib	0.2	23.67 ± 5.79 (1.1)	256.80 ± 27.11 (1.1)
+ erdafitinib	0.5	22.65 ± 5.25 (1.1)	240.19 ± 25.26 (1.2)
+ erdafitinib	1.0	20.77 ± 5.18 (1.2)	229.64 ± 35.28 (1.3)
+ Ko143	1.0	23.80 ± 5.79 (1.1)	20.59 ± 3.66 *** (14.3)
		[nM]	[nM]
SN-38	-	2.93 ± 0.85 (1.0)	83.53 ± 9.80 (1.0)
+ erdafitinib	0.1	2.54 ± 0.49 (1.2)	81.56 ± 12.19 (1.0)
+ erdafitinib	0.2	2.77 ± 0.72 (1.1)	91.97 ± 18.20 (0.9)
+ erdafitinib	0.5	2.45 ± 0.55 (1.2)	74.69 ± 13.69 (1.1)
+ erdafitinib	1.0	2.39 ± 0.58 (1.2)	74.46 ± 14.87 (1.1)
+ Ko143	1.0	3.09 ± 0.98 (0.9)	4.50 ± 0.70 *** (18.6)

FR, fold-reversal. ^†^ IC_50_ values are mean ± SD calculated from dose-response curves obtained from at least three independent experiments using the cytotoxicity assay as described in the Materials and Methods. ^‡^ FR values were calculated by dividing the IC_50_ values of cells treated with a particular therapeutic drug in the absence of erdafitinib or Ko143 by IC_50_ values of cells treated with the same therapeutic drug in the presence of erdafitinib or Ko143. * *p* < 0.05; ** *p* < 0.01; *** *p* < 0.001.

## Supplementary Materials

The following are available online at https://www.mdpi.com/2072-6694/12/6/1366/s1, Figure S1: Expression of ABCB1 in human epidermal cancer KB-3-1 and KB-V-1 cancer cell lines, Figure S2: Expression of ABCB1 in human ovarian cancer OVCAR-8 and NCI-ADR-RES cancer cell lines.
