# Supplementary material for: Erdafitinib Resensitizes ABCB1-Overexpressing Multidrug-Resistant Cancer Cells to Cytotoxic Anticancer Drugs
Source: Cancers (Basel). 2020 May 26;12(6):1366. doi: 10.3390/cancers12061366 (PMC7352346; doi:10.3390/cancers12061366)
Supplement: Supplementary file 1 [file cancers-12-01366-s001.pdf]

Supplementary Materials

Erdafitinib Resensitizes ABCB1-Overexpressing Multidrug-Resistant Cancer Cells to Cytotoxic Anticancer Drugs

Chung-Pu Wu, Tai-Ho Hung, Sung-Han Hsiao, Yang-Hui Huang, Lang-Cheng Hung, Yi-Jou Yu, Yu-Tzu Chang, Shun-Ping Wang and Yu-Shan Wu

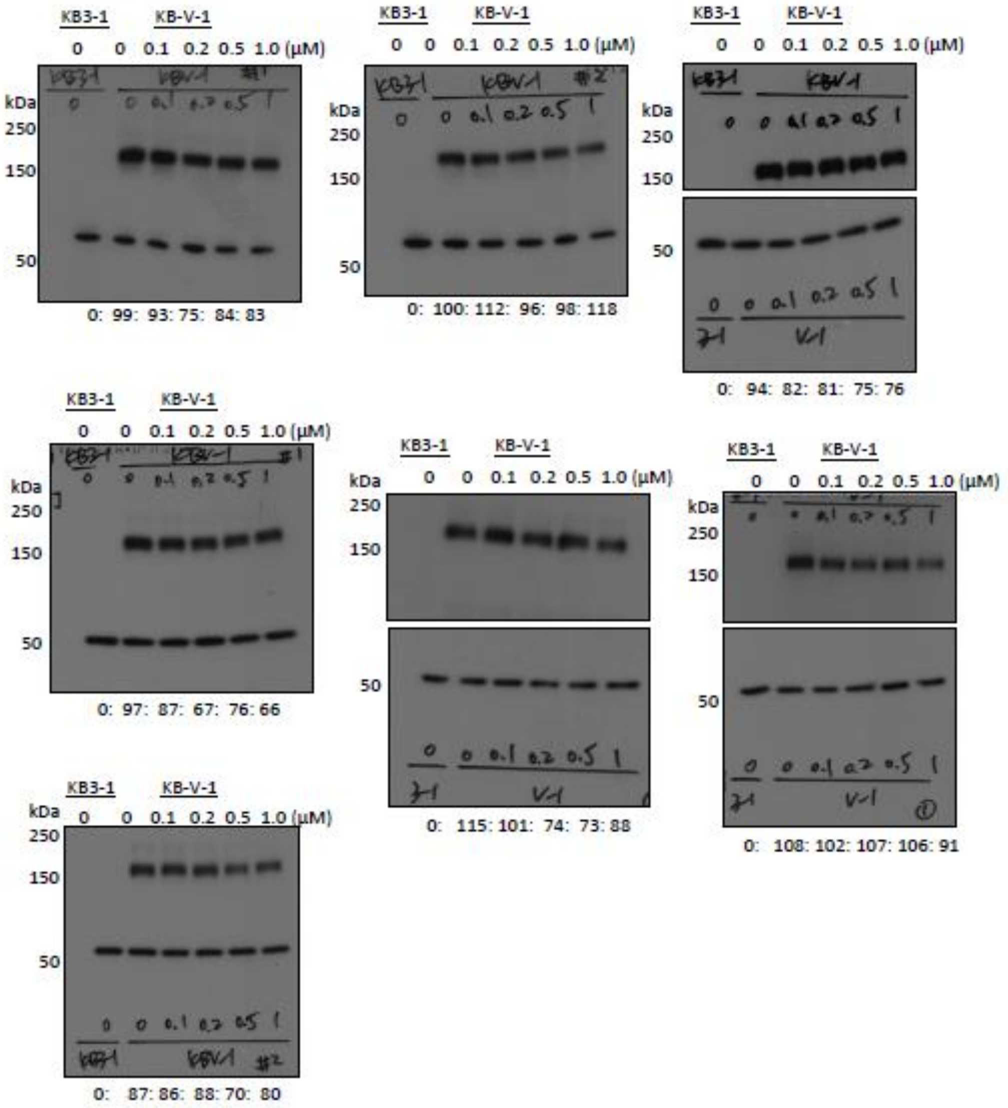

Figure S1. Expression of ABCB1 in human epidermal cancer KB-3-1 and KB-V-1 cancer cell lines.

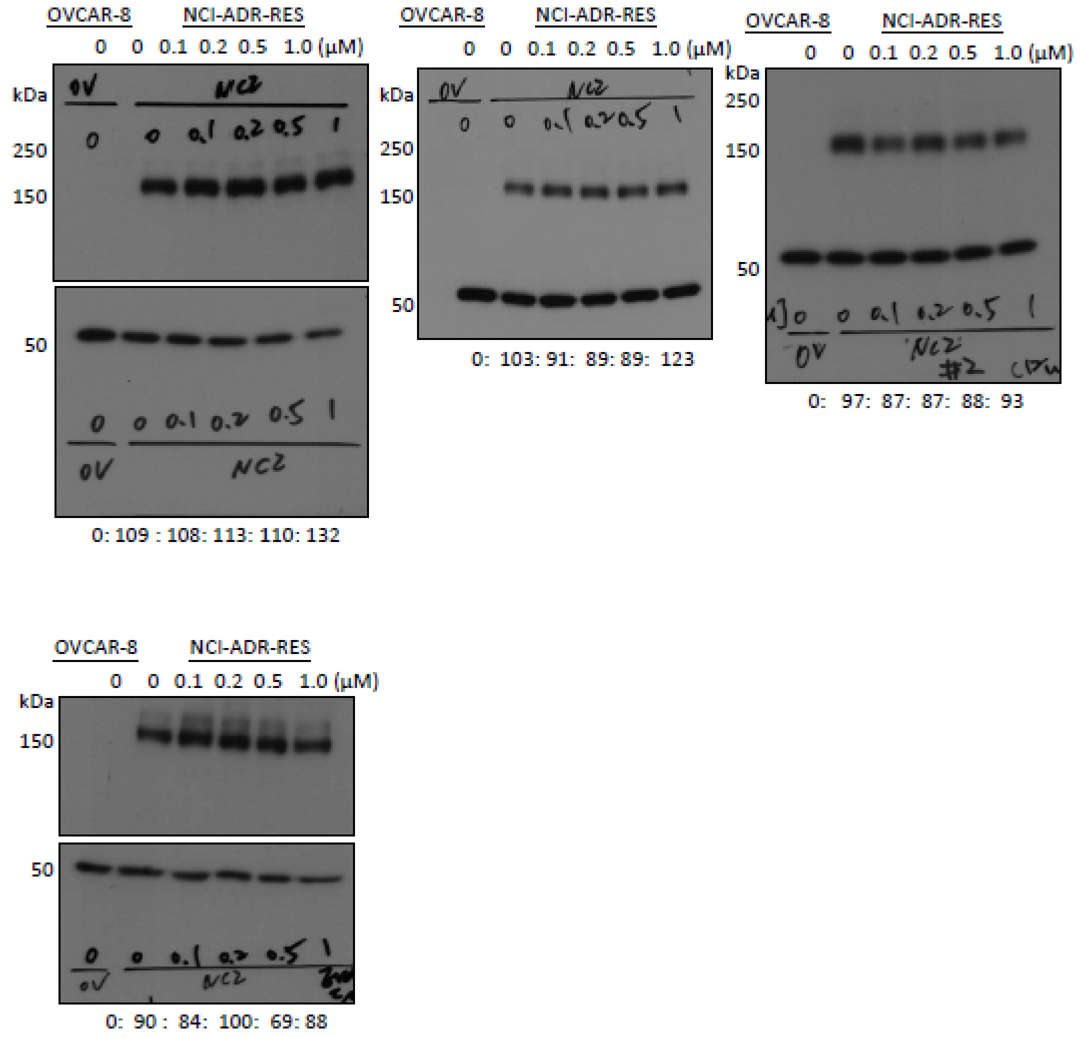

Figure S2. Expression of ABCB1 in human ovarian cancer OVCAR-8 and NCI-ADR-RES cancer cell lines.

© 2020 by the authors. Licensee MDPI, Basel, Switzerland. This article is an open access article distributed under the terms and conditions of the Creative Commons Attribution (CC BY) license (http://creativecommons.org/licenses/by/4.0/).
